# The role of ubiquitin-dependent segregase p97 (VCP or Cdc48) in chromatin dynamics after DNA double strand breaks

**DOI:** 10.1098/rstb.2016.0282

**Published:** 2017-08-28

**Authors:** Ignacio Torrecilla, Judith Oehler, Kristijan Ramadan

**Affiliations:** Cancer Research UK and Medical Research Council Oxford Institute for Radiation Oncology, Department of Oncology, University of Oxford, Roosevelt Drive, Oxford OX3 7DQ, UK

**Keywords:** p97 (vcp/cdc48), DNA double strand breaks, DNA repair, chromatin dynamics, genome stability, CHROMAD

## Abstract

DNA double strand breaks (DSBs) are the most cytotoxic DNA lesions and, if not repaired, lead to chromosomal rearrangement, genomic instability and cell death. Cells have evolved a complex network of DNA repair and signalling molecules which promptly detect and repair DSBs, commonly known as the DNA damage response (DDR). The DDR is orchestrated by various post-translational modifications such as phosphorylation, methylation, ubiquitination or SUMOylation. As DSBs are located in complex chromatin structures, the repair of DSBs is engineered at two levels: (i) at sites of broken DNA and (ii) at chromatin structures that surround DNA lesions. Thus, DNA repair and chromatin remodelling machineries must work together to efficiently repair DSBs. Here, we summarize the current knowledge of the ubiquitin-dependent molecular unfoldase/segregase p97 (VCP in vertebrates and Cdc48 in worms and lower eukaryotes) in DSB repair. We identify p97 as an essential factor that regulates DSB repair. p97-dependent extraction of ubiquitinated substrates mediates spatio-temporal protein turnover at and around the sites of DSBs, thus orchestrating chromatin remodelling and DSB repair. As p97 is a druggable target, p97 inhibition in the context of DDR has great potential for cancer therapy, as shown for other DDR components such as PARP, ATR and CHK1.

This article is part of the themed issue ‘Chromatin modifiers and remodellers in DNA repair and signalling’.

## 1. Introduction

The AAA+ ATPase p97, also known as valosin-containing protein (VCP) in vertebrates (p97/VCP) or Cdc48 in worms and lower eukaryotes (p97/Cdc48), is a central component of the ubiquitin-proteasome system (UPS) [1–3]. p97 uses energy from ATP to remodel (unfold/segregate) ubiquitinated substrates from different macro-complexes and cellular locations, facilitating their proteasome-dependent degradation and/or recycling. As the ubiquitin signal is the main conductor of p97 activity, it is involved in virtually all cellular processes, playing a major role in global protein homeostasis. Specificity towards different ubiquitinated substrates is conferred by p97-adaptor proteins (cofactors), which form various p97 sub-complexes known altogether as the p97 system [3,4]. Through their p97-interacting motifs and (in most cases) ubiquitin-binding domains (UBDs), p97-cofactors bridge the p97 ATPase with specific and mostly ubiquitinated substrates.

As ubiquitin-mediated protein turnover is a fundamental process in chromatin transactions, the p97 system has emerged as an unavoidable genome caretaker [1,5–8]. The p97 system ensures genome stability by processing numerous substrates involved in DNA replication, DNA transcription, DNA repair, mitosis and the cell cycle through a process termed chromatin-associated degradation (CHROMAD). Inactivation of the p97 system leads to hyper-accumulation of ubiquitinated substrates on chromatin resulting in the phenomenon known as protein-induced chromatin stress (PICHROS) [1]. We recommend several recent reviews dealing with the general and chromatin-associated functions of p97 [1,3,4,6,9–11].

Here, we review our current knowledge of the role of the p97 system in the repair of DNA double strand breaks (DSBs), the most cytotoxic DNA lesions. We demonstrate that p97 regulates timing and fidelity of DSB repair at two levels: (i) directly at broken DNA and (ii) on chromatin structure in the vicinity of DSBs. As such, the p97 system represents a unique system that bridges DNA repair and chromatin remodelling machineries to ensure genome integrity after DSB formation.

## 2. DNA double strand break repair

Unrepaired DSBs lead to genome alterations and/or cell death [12,13]. Endogenous DSBs arise after DNA replication fork collapse in proliferative cells or during V(D)J and class switch recombination in lymphocytes, whereas the main exogenous DSB sources are medical ionizing radiation (IR) and some chemotherapeutical drugs. To cope with DSBs, cells activate genome maintenance mechanisms collectively known as the DNA damage response (DDR). The key players in DDR activation are post-translational modifications (PTMs)—mainly phosphorylation, ubiquitination and SUMOylation—which orchestrate the spatio-temporal dynamics of DNA repair and signalling proteins at sites of DSBs. Mutations or polymorphisms in genes that regulate PTMs at sites of DSBs, like those in ataxia-telangiectasia and RIDDLE syndromes, give rise to genome instability and ultimately to diseases manifesting in IR hypersensitivity, cancer susceptibility, immunodeficiency, premature ageing and neurodegeneration.

DSBs are initially sensed by the Ku70/80 heterodimer (Ku), a highly abundant nuclear protein with a rigid double-ring topology and strong affinity (*K*_d_ ∼ 2 nM) for DNA-end structures [14–16]. Upon DSB formation, Ku is immediately threaded onto DNA through the DSB ends with its central cavity encircling duplex DNA. Subsequently, DSBs are repaired mainly by two canonical pathways in eukaryotic cells: Non-Homologous End-Joining (NHEJ) and Homologous Recombination (HR). Although NHEJ is active through the whole interphase, HR is restricted to late S- and G2-phases of the cell cycle [17–19].

**Figure 1.**
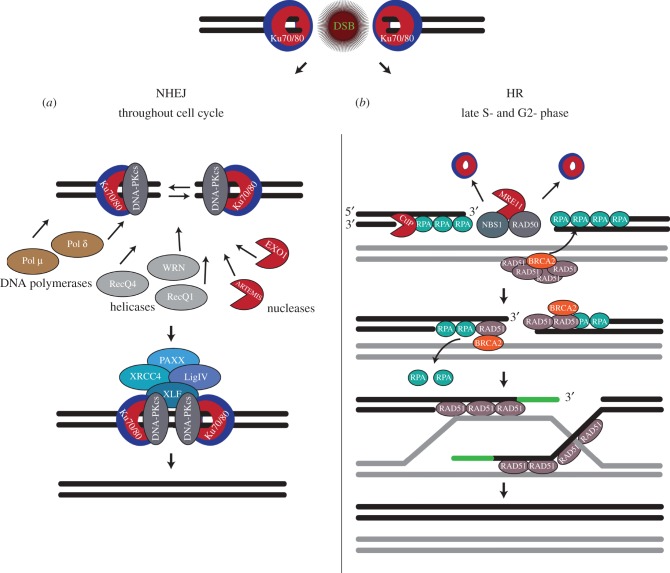
Schematic representation of DSB repair by NHEJ and HR. The broken DNA double strand ends are bound by the Ku70/80 heterodimer, which protects them from extensive end resection. (*a*) Binding and subsequent phosphorylation by DNA-PKcs marks the decision for repair via NHEJ pathway and the broken ends are juxtaposed. The broken ends are mildly processed by different nucleases, helicases and slightly extended by DNA polymerases, to prepare them for re-ligation via the XRCC4-LigIV-XLF complex. This repair mechanism is cell cycle-independent and considered to be error-prone. (*b*) Extensive end-resection by MRE11 (part of the MRN complex) and CtIP nucleases initiates repair via the HR pathway. The 3′-ssDNA overhangs are protected by ssDNA-binding protein RPA, which is consequently replaced by the recombinase RAD51 which is loaded by BRCA2. RAD51 initiates the homology search on the sister chromatid and strand invasion to copy the lost DNA region and thus perform error-free DNA repair. This pathway is mainly executed in late S- and G2-phases of the cell cycle, when a sister chromatid is available.

The canonical NHEJ pathway is the predominant DSB repair pathway in higher eukaryotes and it directly joins any two minimally processed free DNA-ends, irrespective of their sequence [20] or cell cycle stage. As a consequence, this pathway may result in sequence modifications, or even chromosomal rearrangements if the two rejoined ends are unrelated [21]. NHEJ is promoted by DSB-bound Ku, which protects DNA-ends from nuclease degradation and serves as a scaffold for the assembly of the NHEJ repair complex [22,23]. DNA-PKcs is then recruited to Ku and together they form the active DNA-PK kinase complex, which phosphorylates and recruits various downstream substrates that form the NHEJ pathway [16,24–29]. Importantly, DNA-PK also phosphorylates p97 [30], promoting p97 accumulation at DSBs. Depending on the DNA-end topology, additional processing proteins are recruited until DNA-ends are finally re-ligated (figure 1*a*). Further details can be found in [29,31–35].

Contrary to NHEJ, the HR pathway is error-free because it uses a homologous DNA template, typically a sister chromatid, to restore the original sequence at DSB ends [36–40]. Initiation of HR requires preliminary displacement of Ku and extensive 5′–3′ DNA-end resection, performed mainly by the MRN (Mre11, Rad50 and Nbs1) nuclease complex and CtIP nuclease (figure 1*b*). Subsequently, DNA-end resection is further extended by the 5′–3′ exonuclease 1 (EXO1) and Dna2 endonuclease in coordination with the Bloom or Werner helicases (Sgs1 in yeast) [41]. The resulting 3'-ssDNA overhangs are initially coated with phospho-RPA, which is subsequently replaced by the Rad51 recombinase with the assistance of BRCA2 (or Rad52 in yeasts). The ssDNA-Rad51 filaments drive homology search and strand invasion to complete the HR repair pathway.

A feature common to both DSB repair pathways is the initial recognition of free DSB ends by Ku and the association of distinct protein assemblies. While the main proteins of NHEJ and HR have been characterized to varying extents, how their recruitment and especially dissociation are regulated is still unclear.

## 3. p97 in DNA double strand break repair

The first indications of p97 involvement in the DDR arose from the finding that p97 interacts with DNA repair proteins BRCA1 and Werner helicase [42–44] and that p97 is phosphorylated upon DNA damage induction [30]. However, it still took several years to demonstrate a direct role of p97 in DSB repair. Seminal discoveries by two independent laboratories demonstrated that p97 physically associates with DSBs and that inactivation of the p97 system delays DSB repair and hypersensitizes cells to IR [45,46]. Based on these discoveries, a model in which p97 disassembles ubiquitinated substrates from chromatin surrounding DSBs was proposed [5]. Since then, this model has been confirmed by several different laboratories (table 1) [48,49,52,56,57].

**Table 1.** p97 (VCP or Cdc48) cofactors in DSB repair and chromatin remodelling. n.d., not determined; *C. elegans, Caenorhabditis elegans; S. pombe, Schizosaccharomyces pombe; S. cerevisiae, Saccharomyces cerevisiae.*

	p97 cofactor	substrate	species	reference
DSB repair	UFD1, NPL4	K48-ubiquitin conjugates	mammalian cells	Meerang *et al*. [45]
	UFD1, NPL4	L3MBTL1	mammalian cells	Acs *et al*. [46]
	Ufd1	SUMO-conjugates	*S. pombe*	Køhler *et al*. [47]
	Ufd1	SUMO-Rad52	*S. cerevisiae*	Bergink *et al*. [48]
	n.d.	DNA-PKcs	mammalian cells	Jiang *et al*. [49]
	n.d.	KAP1	mammalian cells	Kuo *et al*. [50]
	n.d.	RAD51	*C. elegans*	Ackermann *et al*. [51]
	UFD1, NPL4, FAF1	Ku80	mammalian cells	Van den Boom [52]

	p97 cofactor	interactor	species	reference
chromatin remodelling	n.d.	Irc20	*S. cerevisiae*	Richardson *et al*. [53]
	Ufd1,Npl4, Ubx4, Ubx5, Ubx6, Ubx7	INO80	*S. cerevisiae*	Lafon *et al*. [54]
	UBXD7	MRGX	mammalian cells	Raman *et al*. [55]

Newly formed DSBs are rapidly decorated with mono- and polyubiquitin chains on several substrates generated by ubiquitin ligases like RNF8 and RNF168 [58–64]. Polyubiquitin chains with K63-ubiqutin and K27-ubiquitin chains mostly serve as recruitment platforms for DDR proteins, whereas those with K48-ubiquitin chains are mostly signals for protein removal and proteasome-dependent degradation [5,65,66]. p97 complexed with the cofactors Ufd1-Npl4 (p97^Ufd1-Npl4^) recognizes the ubiquitin signal and associates with sites of DSBs [45,46]. Despite the key role of RNF8, the recruitment of p97^-Ufd1-Npl4^ only partially depends on it, suggesting that additional E3 ligases and/or PTMs like SUMOylation might regulate p97 recruitment. Indeed, ubiquitination by the SUMO-targeted E3-ubiquitin ligase (STUbL) RNF4, as well as SUMOylation of Rad52 in yeast, also engage p97 at DSBs [48,50]. Once recruited, p97 physically interacts with and removes different ubiquitinated proteins like L3MBTL1, Ku and KAP1, and allows the loading of other proteins like 53BP1, BRCA1 and Rad51 [45,46,50,52]. p97 thus facilitates both DSB repair pathways and regulates the organization of chromatin structure surrounding DSBs.

### (a) p97 (VCP) in non-homologous end-joining

Loss of the p97^-Ufd1-Npl4^ complex compromises NHEJ activity [45,52]. One mechanism through which p97 facilitates NHEJ is by segregating the ubiquitinated polycomb protein L3MBTL1 from DSB sites, which in turn enables recruitment of 53BP1 (see below) [46,67–69].

**Figure 2.**
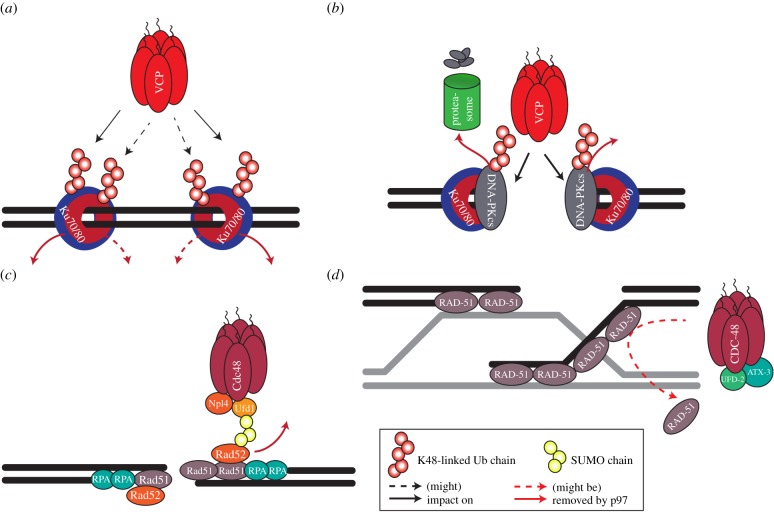
The role of p97 in DSB repair. (*a*) The Ku complex becomes trapped on the re-ligated DNA post-NHEJ. To be unloaded, Ku80 (blue) is K48-ubiquitinated and removed by the action of p97 ATPase activity (VCP in vertebrates). A similar mechanism is possible but not yet shown for Ku70 (red). (*b*) DNA-PKcs is tagged with K48-linked ubiquitin chains. This serves as a signal for p97 to unload it from the DNA and deliver it for proteasomal degradation. (*c*) Cdc48 (p97 in yeast) recognizes SUMOylated Rad52, a loader of Rad51, via a SIM in Ufd1. Thus, Cdc48 removes Rad52 and avoids hyper-loading of Rad51 onto the ssDNA. Cdc48 therefore prevents hyper-recombination events during HR. (*d*) The CDC-48 (p97 in *Caenorhabditis elegans*) system, in collaboration with the UFD-1-NPL-4 core adaptor complex, the deubiquitinating enzyme ataxin-3 and the E4-ubiquitin enzyme UFD-2, is involved in disassembly of RAD-51 filaments. A role of mammalian UFD-2 homologues, UBE4A and UBE4B, in the DDR has yet to be addressed.

NHEJ completion also requires p97. When DNA-ends are rejoined, the ring-shaped Ku complex gets sterically interlocked on the DNA molecule (figure 2*a*). If not removed, post-repair persistence of trapped Ku would interfere with DNA replication and transcription [70,71]. However, the rigidity of Ku impedes it from being opened for release like other DNA clamps such as PCNA [72]. Instead, segregation of Ku requires profound structural remodelling performed by p97 [73], which depends on prior conjugation of Ku80 with K48-ubiquitin chains [52,73]. Inactivation of p97 results in the accumulation of ubiquitinated Ku80 and K48-ubiquitin chains at the sites of DSBs [5,45,52]. This was demonstrated by mass spectrometry and by *in vivo* and *in vitro* analysis of Ku80 recruitment to DSBs in mammalian cells and *Xenopus* egg extract [45,52,56]. Although not shown, it is conceivable that p97 might be also required for extraction of the Ku70 subunit. Ku70 ubiquitination has been shown, but it is not clear whether this modification targets it for p97 segregation [32,56,74].

Other roles for p97 in NHEJ are expected, particularly during initiation because p97-depleted human cells exhibit reduced NHEJ [45]. Of note, p97 can interact directly with ubiquitinated DNA-PKcs to promote its proteasomal degradation (figure 2*b*) [49].

### (b) p97 (VCP) in homologous recombination

Besides its involvement in NHEJ, p97 also enables HR initiation and progression. In mammalian cells, p97 depletion largely attenuates HR after treatment with DSB-inducing agents that act either independently of cell cycle (like IR or Zeocin) or specifically during S-phase by inducing replication fork collapse (like the chemotherapeutic drug camptothecin) [45,52]. The most deleterious effects observed on HR following p97^-Ufd1-Npl4^ inactivation are impaired DNA-end resection and a consequent broad decrease in loading of HR-associated proteins phospho-RPA, Rad51 and BRCA1, combined in some instances with an abnormal nuclear accumulation of Rad51, observable by high-resolution microscopy [48]. Defects in DSB end resection are most probably due to hyper-accumulation of Ku at DSB sites which, as mentioned, prevents DNA-end resection, the main HR commitment step. Early eviction of Ku in S/G2-phases releases the brake on HR and, notably, has been shown to be driven by p97, indicating that p97 contributes to the DNA repair pathway choice by favouring commitment to HR. Repair pathway choice after DSB formation is a crucial initial decision that affects the fidelity and speed of repair.

### (c) p97 (VCP) in repair pathway choice

As discussed, Ku is removed by p97 at both pre-HR and post-NHEJ repair stages. The selective action of p97 may reflect cell cycle and/or repair stage-dependent differences of various ubiquitin ligases with non-redundant functions. For example, the early ubiquitination of Ku involves RNF138 [75], an E3-ubiquitin ligase that operates mainly during S/G2 cell cycle phases. This notion could be extended to the segregation of other DNA repair proteins like KAP1 [50], which is signalled for p97-dependent proteasome degradation by RNF4, another ubiquitin ligase that accumulates in S/G2 phase. In agreement with this, numerous lysines in Ku have been identified to be either ubiquitinated [56] or accessible for potential ubiquitination [76], and several other ubiquitin ligases are likewise able to promote K48-ubiquitin chain formation to degrade DNA-bound Ku80, including RNF8 [77] and cullin-RING ubiquitin ligases [56,73,74]. It is therefore possible that several ubiquitin ligases, regulated by alternative pathways, conjugate ubiquitin to Ku to trigger its removal. In this regard, an Fbxl12-containing Skp1-Cullin-F-box (SCF) complex is also able to promote the conjugation of K48-ubiquitin to Ku80 in *Xenopus* egg extract [73].

### (d) p97 (Cdc48) in non-mammalian homologous recombination

Rad51 loading is primarily mediated by BRCA2 in metazoans and by Rad52 in yeasts. A control mechanism in *Saccharomyces cerevisiae* to restrain Rad52, and thus control Rad51 loading, involves p97/Cdc48^-yUfd1^ binding to SUMOylated Rad52 through a SUMO interacting motif (SIM) present in yeast Ufd1 (yUfd1) [48]. This binding counterbalances the interaction between Rad52 and Rad51 to suppress spontaneous recombination events (figure 2*c*). Despite the importance of SUMOylation in Rad51 loading in metazoan cells [78] and the identification by mass spectrometry of SUMOylated BRCA1/BRCA2 [79,80], a similar mechanism in mammalian cells seems unlikely, given that the mammalian Ufd1 apparently lacks SIMs [81] and that the mammalian Rad52 orthologue has a less significant role in HR.

Experiments in *Caenorhabditis elegans* provided indications that following high doses of IR, p97/Cdc48 may be relevant for the coordination between ongoing HR repair and activation of apoptosis [51,82,83]. p97/Cdc48 was able to disassemble RAD-51 filaments and to interact and stimulate the activity of proapoptotic UFD-2, reinforcing the position of p97/Cdc48 in the key decision between DNA repair and apoptosis [51] (figure 2*d*). Whether mammalian Ufd2 orthologues UBE4A and UBE4B are also involved in apoptosis control is unclear, but it was shown that UBE4B regulates levels of p53 [84,85].

## 4. The role of p97 in double strand break-induced chromatin remodelling

In order to ensure proper gene expression and genome stability, eukaryotic nuclear DNA combines with histones and other proteins enabling the formation of highly organized chromatin structures. Nucleosomes are the core particles of chromatin, consisting of an approximately 146 base-pair-long DNA segment coiled around a histone octamer, which consists of two copies of both H3 and H4 histone proteins and two H2A/H2B histone dimers. A fifth histone, H1, links consecutive nucleosomes, which are further bundled together into three-dimensional chromatin fibres with varying layers of compaction. The final chromatin architecture is dictated by nucleosome variability, which results from incorporating different histone core variants (such as H2A variants H2AX and H2AZ) and from the epigenetic markers carried on them. These markers are modulated by the activity of many different enzymes and read by sensor proteins coupled to effectors [86–88].

Upon DNA damage, a rapid chromatin adaptation at sites of DSBs and surrounding chromatin ensures proper DNA repair [89]. The global process known as chromatin remodelling facilitates efficient access to nucleosomal DNA, mediates signalling and controls the activity of repair proteins. The chromatin landscape is chiefly organized by the action of histone PTM enzymes and ATP-dependent remodelling complexes, which catalyse the chemical modification of epigenetic markers and the physical rearrangement of nucleosomes, respectively. This leads to changes in chromatin-associated proteins that regulate the kinetics of DSB repair and also inhibit RNA transcription [88]. For example, the gene transcription co-repressor KRAB-associated protein 1 (KAP1) is phosphorylated by the ATM kinase and is rapidly recruited to chromatin surrounding DSBs, where it represses transcription and makes chromatin more accessible for repair [90]. The importance of chromatin remodelling in the DDR [89,91] along with recent discoveries regarding the activity of p97 in this context [11,92] are spurring wider attention to the influence of p97 on chromatin reorganization during DSB repair.

### (a) p97 regulates DNA damage-induced histone modifications

**Figure 3.**
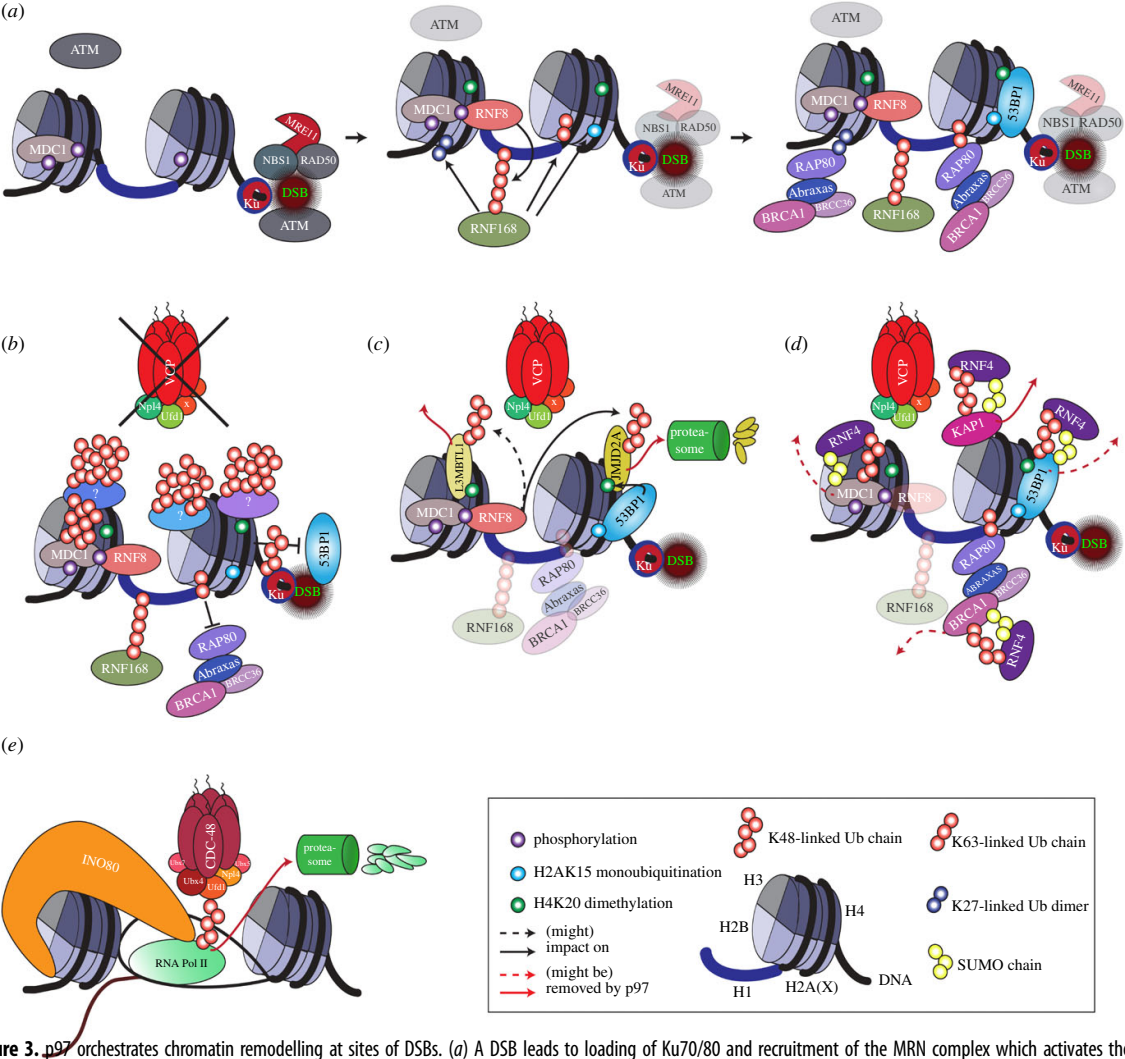
p97 orchestrates chromatin remodelling at sites of DSBs. (*a*) A DSB leads to loading of Ku70/80 and recruitment of the MRN complex which activates the ATM kinase which phosphorylates H2AX. This phosphorylation mark is recognized by MDC1 which itself is phosphorylated by ATM. RNF8 binds to phosphorylated MDC1 and ubiquitinates histone H1 with K63-linked ubiquitin chains. This recruits RNF168 which initiates the ubiquitination on histone H2A(X) on lysine13 and lysine15. Subsequently, this leads to ubiquitin-dependent recruitment of the two tumour suppressor proteins 53BP1 and BRCA1. (*b*) Inactivation of the p97 system leads to hyper-accumulation of ubiquitinated substrates at DSBs, especially of K48-linked chains, on the Ku complex, L3MBTL1 and most probably on other yet to be confirmed substrates. Defective VCP activity at DSBs leads to abolition of 53BP1 and BRCA1 recruitment which affects both DSB repair pathways. (*c*) VCP facilitates 53BP1 binding to histone H4 methylated at lysine 20, by removing L3MBTL1 and possibly JMJD2A from these sites, both tagged with K48-linked ubiquitin chains. JMJD2A is targeted for proteasomal degradation. (*d*) VCP collaborates with the STUbL RNF4. KAP1 is removed from chromatin by VCP upon SUMOylation-dependent ubiquitination by RNF4. Additional identified RNF4 targets include MDC1, BRCA1 and 53BP1, and all of them are potential VCP substrates. (*e*) INO80 and Cdc48 collaborate to unload arrested RNA polymerase II complex. Arrested and ubiquitinated RNA polymerase II is unloaded from chromatin by INO80 and the Cdc48 system and finally degraded by the proteasome. Note that p97 is also called VCP in higher eukaryotes, and Cdc48 in yeast and worms.

DDR induction results in the manifestation of an extensive array of specific covalent PTMs on structural chromatin proteins to facilitate repair, including methylation, acetylation, phosphorylation, ubiquitination and SUMOylation [93–95]. Specifically, ubiquitination signalling has been recently established as a fundamental DDR constituent for the time- and spatially coordinated mobilization of DNA repair proteins [96,97]. Various ubiquitin ligases, mainly RNF8 and RNF168, initiate the ubiquitination cascade at DSB sites (figure 3*a*). RNF8 first marks histone H1 with K63-linked ubiquitin chains [98]. RNF168 recognizes this modification through its UDM1 ubiquitin-binding domain [61,69] and subsequently ubiquitinates histones H2A and H2AX [99,100]. This second modification is also recognized by RNF168 through another UBD termed UDM2, which further anchors RNF168. Ubiquitination of other proteins follows. Although the topology of these ubiquitin chains is imprecise, RNF168 is able to add mono- and K27-linked ubiquitin [66,100], while RNF8 mainly produces K48-ubiquitin and K63-ubiquitin chains [45,77,101,102]. While we understand the formation of ubiquitination signals and attraction of DDR proteins to some extent, less is known about their functional consequences and the fate of ubiquitin-modified proteins.

p97 docks to ubiquitin chains at the sites of DSBs [45,46] through the UBDs borne by core p97-adaptors, chiefly Ufd1 and Npl4 [45]. Once at the sites of DNA damage, p97 orchestrates the segregation of ubiquitinated substrates, resulting in the attenuation of the global ubiquitin signals at sites of DNA damage [45,57]. This function becomes evident in cells with abolished p97 activity, which exhibit hyper-accumulation of ubiquitin chains at DSBs (especially those with K48-ubiquitin chains), impairment of NHEJ and HR repair pathways, and attenuated recruitment of DNA repair and signalling proteins Rad51, 53BP1 and BRCA1 [45] (figure 3*b*). While this clearly indicates that p97 hierarchically removes DDR proteins from the dynamic DNA repair complexes, only a few unequivocal examples of p97 substrates have been reported, despite the great interest of this matter.

53BP1 is one of the essential DSB-signalling proteins whose function in DSB repair relies on p97 (figure 3*c*). 53BP1 binding to chromatin is mediated through its ubiquitination-dependent recognition UDR and Tudor motifs recognizing, respectively, H2A monoubiquitination (H2A-Lys-15Ub) [103] and H4 methylation (H4-Lys-20me2) [104] marks. Although the first mark is DSB-specific generated by RNF8/RNF168, the second one is very abundant even in unstressed cells and usually occupied by other Tudor domain-containing proteins L3MBTL1 and JMJD2A/B. In order for 53BP1 to gain access to sites of DSBs, L3MBTL1 must be ubiquitinated and degraded in an RNF8- and p97-dependent manner. Removal of L3MBTL1 exposes H4-Lys-20me2, allowing recruitment of 53BP1 to DSB sites [46,105]. JMJD2A/B extraction also depends on RNF8 and proteasome [67] but a role for p97 here was unaddressed.

Marks for proteasomal degradation are not limited to the activity of conventional E3-ubiquitin ligases. Protein SUMOylation has emerged as an additional control mechanism for protein turnover, acting in concert with ubiquitination through the SUMO-targeted ubiquitin ligase (STUbL) RNF4 (figure 3*d*). RNF4 interacts with p97 and the absence of either protein leads to stabilization of KAP1 [50]. This begs the question of whether p97 also participates in the removal of the other known SUMOylated RNF4 targets like MDC1, 53BP1, BRCA1 and RPA [78]. Rad22 (RAD52 in mammalians) has been identified as one of the STUbL/p97/Cdc48 substrates [47,81]. As mentioned, yeasts rely on additional mechanisms for protein degradation through a SUMO interacting domain (SIM) present in yeast Ufd1 that targets p97/Cdc48 to SUMOylated substrates.

Besides regulating histone modifications after DSBs, p97 also influences chromatin dynamics and DNA repair pathway choice by regulating KAP1. IR induces the formation of discrete phospho-KAP1 (pKAP1) foci at DSBs, and this happens predominantly in cells in G0/G1-phase of the cell cycle [50]. Mechanistically, pKAP1 concomitantly promotes NHEJ and impedes HR repair by blocking the loading of BRCA1 [50]. Removal of KAP1 foci during S/G2-phases by RNF4 and p97 thus favours HR repair. Indeed, direct interaction between p97 and RNF4 is required *in vivo* for the removal of pKAP1 and subsequent loading of BRCA1 and RAD51. A remaining question is the identity of the p97 cofactor(s) involved in this process.

### (b) Cooperation between the p97 system and chromatin remodelling complexes

ATP-dependent chromatin remodelling complexes (CRC) tailor chromatin configuration by employing energy from ATP hydrolysis to disrupt DNA–histone contacts and mobilize (slide, twist, loop and evict) nucleosomes along DNA or alter their composition (exchange histones) [106]. All CRCs contain a core catalytic subunit harbouring a DNA-dependent ATPase/helicase domain and one regulatory domain whose identity defines four major families of CRCs: INO80, ISWI, Mi-2/CHD and SWI/SNF. Catalytic subunits are encoded by a minimum of 30 different genes in mammals (nine in yeast) [107,108]. Additional subunits conferring distinct biological functions can then associate with the ATPase subunit, resulting in a variety of complexes within each family [107,109,110]. Although primarily studied in yeast models, CRCs are evolutionary conserved with homologous subunits being present across eukaryotes. Recent reviews provide details of the structure and functions of CRCs [89,109,111–113]. Here, we focus on the physical and functional interaction between CRCs and the p97/Cdc48 system.

DDR-associated changes in chromatin commit all four CRC families to cooperatively rearrange nucleosome density and composition around DSBs and to modulate DNA repair proteins. For example, the recruitment of Ku is fostered by ACF1 (ISWI family) [112], whereas HR-promoting DNA resection by the MRN nuclease complex is further enhanced by several complexes including INO80, the histone-acetylase Tip60/NuA4 (SWR1) and BRG1 (SWI/SNF) [111,114], the latter also facilitating pRPA/RAD51 replacement [115,116]. H2A.Z/H2B and H2A/H2B dimer exchange is controlled by opposing roles of INO80 and SWR1, and γH2AX is maintained by INO80 [109,111,117–119].

### (c) Interaction of chromatin remodelling complexes with the ubiquitin-proteasome system

DSB signalling also intertwines with CRCs. Several CRCs are phosphorylated by ATM (BRG1, BAF180 [120,121]), and some CRCs promote deacetylation and checkpoint activation [122–124]. Here as well, the ubiquitination signalling cascade induced by DSBs is taking centre stage. The ubiquitin ligase RNF168 interacts with the catalytic subunit SNF2H/SMARCA5, and the activities of both ISWI and CHD4 complexes are required for RNF168 function [122,125–128]. Previous monoubiquitination of histone H2B is one factor supporting the recruitment of SNF2H itself [129–131]. Links between CRCs and the UPS are further represented by the metazoan INO80 complex. Upon interaction of INO80 with the proteasome, its UCH-L5/Uch37 subunit exhibits deubiquitinating activity on K48-ubiquitin chains [132,133]. It is noteworthy that UCH-L5/Uch37 can also be found in the eukaryotic 26S proteasome [132,134]. Interestingly, UCH-L5 and INO80 ATPase, along with the proteasome and the RNA polymerase II machinery, has been associated with progression of Alzheimer's disease [135].

Affinity-capture screenings and mass spectrometry analysis suggest that the p97/Cdc48 system physically interacts with numerous CRCs. For example, yeast p97/Cdc48 and several p97-cofactors (Ubx4, Ubx5, Ubx6 and Ubx7) and INO80 components (Ino80, Arp5 and Arp8) were co-precipitated in reciprocal pull-down assays [54]. Indeed, during transcription arrest, p97/Cdc48-Ubx7 was shown to form a ternary complex with INO80 and polyubiquitinated Rpb1, the largest subunit of RNA polymerase II essential for polymerase activity (see below) [54]. A physical interaction between p97/Cdc48 and Irc20 was discovered to regulate transcription [53]. Irc20 and its mammalian homologues SHPRH and HLTF are ATP-dependent chromatin remodelling helicases with E3-ubiquitin ligase activity. Although they are involved in DNA repair and replication [136–138], whether p97/Cdc48 interacts with Irc20 in that context is unknown. In non-stimulated human cells, some UBXD-containing p97-cofactors exhibit significant interaction with subunits in the SWI/SNF, NurD and INO80 complexes [55], like UBXD7 with MRGX (TRAAP/NuA4 sub-complex). Although the functions of these detected interactions between p97/Cdc48 and CRC components are generally unknown, they reveal that the ATPase activities in each system may be complementary during chromatin remodelling. A challenge for future research will be to uncover the functional significance and to determine how their activities are coordinated at DSB sites.

### (d) Removal of arrested RNA polymerase II by interaction of INO80 and UPS systems

A remarkable example of concerted action between CRC and p97/Cdc48 systems is found during stalled RNA transcription in *Saccharomyces cerevisiae* [54,139]. RNA transcription frequently stalls at sites of DNA damage [140,141], creating an obstacle to DNA replication and repair machineries. In these conditions, the chromatin-bound subunit of RNA polymerase II, Rpb1, undergoes polyubiquitination [142–144] followed by p97/Cdc48-dependent extraction and proteasomal degradation [139]. A report by Lafon *et al*. [54] established that the INO80 complex is also required and coordinates with p97/Ccd48 in RNA polymerase II extraction (figure 3*e*). INO80 and p97/Cdc48 form an inter-complex interaction network together with p97-cofactors Ufd1, Npl4, Ubx4 and Ubx5 that engages in a ternary complex with ubiquitinated Rpb1 to promote its proteasomal degradation. Ubx7 appears to be the main p97/Cdc48 cofactor stabilizing this structure. Cells lacking INO80 or p97/Cdc48 exhibit an aberrant accumulation of polyubiquitinated RNA polymerase II on chromatin. This scenario places INO80 as a player in UPS-dependant protein turnover on chromatin. It is plausible that INO80 and p97/Cdc48 converge at a stage in which the translocase activity of INO80 may be important to disrupt the contacts between Rpb1 and chromatin, with the segregase activity of p97/Cdc48 required to extract it. Consistent with this model, INO80 and p97/Cdc48 exhibit synergistic effects in promoting DNA replication and cell viability after treatment with different DSB-inducing agents, including treatments with HU, MMS or Zeocin [54]. A pertinent question is whether an analogous mechanism is present also in mammalian cells.

## 5. Conclusion

The p97 system has recently enlarged the list of genome caretakers required to perform the repair of DSBs. AlthoughCHROMAD is still far from being completely understood, we can now safely claim that p97-orchestrated CHROMAD is an essential process during DSB repair. To be specific, various and mostly ubiquitinated proteins involved in many aspects of DSB repair, from initiation to completion, have to be spatio-temporally removed by p97. p97 hierarchically removes chromatin-bound proteins from sites of broken DNA as well as from surrounding chromatin, as illustrated by its removal of L3MBTL1, the Ku heterodimer and KAP1. In addition, the p97 system cooperates with CRC at DSBs to remodel the chromatin landscape and facilitate protein degradation at nearby sites of replication and transcription, illustrated by the mechanistically described cooperation with INO80.

Many questions remain open and future research should resolve the composition, interactions and functional differences of the diverse p97-cofactor sub-complexes as well as identify additional substrates. In addition, investigating the interactions between the p97 system and CRCs could be equally promising at resolving additional regulatory mechanisms.

Answers to these questions will help us to better understand the processes underlying genome stability and underscore the importance of the p97 system as a druggable target for cancer therapies, based on the inactivation of the DDR.
